# The Detection of Malingered Amnesia: An Approach Involving Multiple Strategies in a Mock Crime

**DOI:** 10.3389/fpsyt.2019.00424

**Published:** 2019-06-17

**Authors:** Stefano Zago, Emanuela Piacquadio, Merylin Monaro, Graziella Orrù, Erika Sampaolo, Teresa Difonzo, Andrea Toncini, Eugenio Heinzl

**Affiliations:** ^1^U.O.C. Neurologia, IRCSS Fondazione Ospedale Maggiore Policlinico di Milano, Milano, Italy; ^2^Department of General Psychology, University of Padova, Padova, Italy; ^3^Department of Surgical, Medical, Molecular & Critical Area Pathology, University of Pisa, Pisa, Italy; ^4^IMT School for Advanced Studies Lucca, Lucca, Italy; ^5^Dipartimento di Medicina Veterinaria, Università degli Studi di Milano, Milano, Italy

**Keywords:** amnesia, crime, mock crime, malingering detection techniques, malingering

## Abstract

The nature of amnesia in the context of crime has been the subject of a prolonged debate. It is not uncommon that after committing a violent crime, the offender either does not have any memory of the event or recalls it with some gaps in its recollection. A number of studies have been conducted in order to differentiate between simulated and genuine amnesia. The recognition of probable malingering requires several inferential methods. For instance, it typically involves the defendant’s medical records, self-reports, the observed behavior, and the results of a comprehensive neuropsychological examination. In addition, a variety of procedures that may detect very specific malingered amnesia in crime have been developed. In this paper, we investigated the efficacy of three techniques, facial thermography, kinematic analysis, and symptom validity testing in detecting malingering of amnesia in crime. Participants were randomly assigned to two different experimental conditions: a group was instructed to simulate amnesia after a mock homicide, and a second group was simply asked to behave honestly after committing the mock homicide. The outcomes show that kinematic analysis and symptom validity testing achieve significant accuracy in detecting feigned amnesia, while thermal imaging does not provide converging evidence. Results are encouraging and may provide a first step towards the application of these procedures in a multimethod approach on crime-specific cases of amnesia.

## Introduction

Crime-related amnesia is a controversial problem and the subject of a prolonged debate (1–5). It has been observed that offenders report total or partial amnesia regarding a violent homicide in a range of 10% to 70% of the cases, depending on the literature reviewed (5–8).

Tracing the history of the phenomenon, the interesting stories of crime-related amnesia of Rudolf Hess (9) and Guenther Podola (10) can be found, where amnesia seems easy to pretend and difficult to disprove, and it arises as part of a defense strategy using loss of memory as mental incompetency to stand trial (11).

Even in the recent past, some courts expressed the view that amnesia is an important point to consider when answering the question about whether or not the defendant can receive a fair trial (12).

Most clinicians, forensic experts, and judges though are skeptical about the development of such an authentic crime-related amnesia. Notwithstanding this marked skepticism, researchers demonstrate that, apart from malingering, some cases of crime-related amnesia are genuine and could be attributed to a range of temporary brain dysfunctions. Acute alcohol and drug intoxications (13, 14), sleep disorders (15, 16), psychotic episodes (17), or dissociative states in traumatic and/or under stressful events (6, 18) are some examples. In particular, it has been hypothesized that dissociative states relate to neurotransmission and neuroendocrine dysregulations, underlying an organic cause. However, as Pyszora and colleagues argued, crime-related amnesia has often a psychogenic origin (19, 20), a condition that could determine an adverse effect in attention and in the consolidation of memories related to crime (21). Yet, a partial or complete recovery of memories is possible (19).

According to many adversarial criminal systems (US, UK, and most European countries), amnesia for crime, as an isolated reported symptom, which is not clustered within another neurological or psychiatric disorder, cannot be the basis of any mental insanity or reduced capacity claim. However, when associated with a neurological or psychiatric disorder, amnesia may call for additional safeguards to guarantee a fair trial (11, 22, 23). According to the Italian Penal Code, this is the case when the defendant is suffering from a genuine amnesia (e.g., from a neurological illness), preventing his recollection of the real fact as it is unfolded during the crime itself. Consequently, the defendant’s amnesia would give the prosecutor an improper advantage in the legal confrontation. This improper advantage would undermine the legal basis of the adversarial system, which requires equal opportunity of prosecution and defense in front of the judge. When loss of memory appears to be temporary, the trial could be deferred for a reasonable period of time to allow improvements of the defendant’s amnesia. In Italy, for instance, these cases undergo periodic reassessments (usually every 6 months).

Given that fraudulent claims about amnesia are easy to be feigned, it is important to evaluate whether such amnesia is genuine or made up.

Forensic experts require a significant clinical and testing expertise in order to accurately evaluate amnesic disorders in criminal proceedings. Hence, a deep knowledge of neurobiological correlates of memory is needed. The same understanding is required for its processes, various amnesic syndromes, and their underlying organic or psychogenic causes (24). During the examination, it is important to have a thorough examination of offenders, reconstructing the history of their memory disorder, interviewing them, analyzing the circumstances of the crime in all its details, and inquiring into all the situations that preceded and followed the violent performance. Along with a neuropsychological standard evaluation including memory tests, a set of neuroimaging [Computerized axial tomography (CAT) and Magnetic resonance imaging (MRI) scans] and neurophysiological [Electroencephalography (EEG)] acquisitions may highlight lesions related to diseases or anomalous brain functioning. On a practical level, this preliminary knowledge and tools themselves could lead to a solution in some cases.

However, forensic specialists can also supply these traditional applications by using different tools specifically designed to detect lie and autobiographical memory veracity. Polygraph, event-related potentials (ERPs), functional magnetic resonance imaging (fMRI), facial expression analysis, thermal imaging, or neuropsychological procedures such as symptom validity testing (SVT), autobiographical Implicit Association Test (a-IAT), or kinematic technique have all been proposed as potential methods to detect genuine crime amnesia (25–27).

Despite the extended literature on laboratory studies regarding lie and memory detection, published single case reports on defendant’s amnesia where these emerging techniques are applied are very few. Single-case application is, to our knowledge, limited to some studies with polygraph, ERPs, and SVT. For example, Jelicic (28) applied SVT in a case of a 29-year-old man who stabbed his girlfriend to death while claiming to have forgotten the details of the crime. Using SVT, the author argued that this was a circumstance where malingering occurred.

An alternative possibility to study feigned amnesia is the preliminary application of these techniques in mock crime experiments [e.g., Refs. (29–33)]. However, only in the study of Giger and colleagues was a more realistic homicide scenario built in which the participants had to hit with great force the victim. In the other studies, subjects were instructed to perform petty thefts of things or money. A limitation of the mock crime studies has been raised by Merckelbach et al. (29) that pointed out the little ecological validity of this kind of experimental design. For example, there is no doubt that, in cases of criminal amnesia, there are higher levels of emotional arousal (34), which are impossible to replicate in mock crime experimental conditions. It will therefore be necessary in the future, when feasible, to directly assess the compatibility of laboratory data (e.g., mock crime) and real-life data of offenders.

Currently, a clear line of demarcation between experimental analysis and real practical forensic application has yet to be defined. Nowadays, it is possible to see these techniques as a useful support to the clinical analysis of crime-related amnesia. Moreover, it is crucial to satisfy the Daubert Standard Criteria within such well-established practices. The U.S. Supreme Court, in Daubert v. Merrell Dow, outlined six criteria for the federal judge to consider when determining the admissibility of evidence (35). These criteria govern the acceptability of scientific tests based on the percentage of reliability of a technique, the publication of relevant studies in peer-reviewed journals, and the general consent among the scientific community.

The purpose of this paper is to evaluate the efficacy of three emerging techniques in evaluating crime-related amnesia, i.e., thermal imaging, kinematic analysis, and SVT, in a group of subjects invited to simulate, or not, amnesia following a mock homicide. The choice of these three techniques is motivated by the fact that they can be administered in a multi-method approach in a simple and non-interfering way. Thermal imaging is based on autonomic responses, while kinematic analysis and SVT are based on cognitive elaboration. A brief review of these techniques is reported below.

## Three Emerging Techniques to Evaluate Crime-Related Amnesia

### Thermal Facial Imaging

Thermal infrared imaging is a widely used technique to measure heat emission from the body, transformed into an infrared band of the electromagnetic spectrum (36). Body temperature, and in particular facial temperature, reflects the activity of the autonomic nervous system during the natural exposition to social interaction and communication (37). For this reason, psychophysiologists are interested in the measurement and recording of these bodily changes. An interesting application of thermal infrared imaging is in the lie detection field. In particular, researchers analyzed facial skin surface temperature (SST) in deceptive and non-deceptive participants while performing a Concealed Information Test (CIT) (38–41). During the arousal, an increase in SST in the periorbital region around the eye and the nose was found. This may suggest a plausible association with specific emotions. Generally, data showed that deceptive subjects had a higher temperature in these regions compared to non-deceptive ones. For example, in the study of Pavlidis et al. (38), 83% of the participants were correctly recognized as mentors (75%) or innocents (90%) by the analysis of thermographic images. For the same subjects analyzed with the polygraph, the accuracy was lowered up to 70%.

A simple objection of thermal imaging application is that an increase in blood flow in the periorbital zone is also associated with prolonged stress, and a stressed person could be wrongly judged as guilty.

To our knowledge, there are no studies analyzing thermal imaging results on crime-related amnesia.

### Kinematic Technique

A recent technique, also referred to as *kinematic technique*, has been introduced by Monaro et al. (42, 43) to detect fake responses regarding identity. It is based on recording motor response of subjects involved in a computer task while using a mouse. The mouse movement analysis may be used as an implicit measure to investigate the cognitive processes underlying a task (44), including the cognitive processes underlying the deception production (45). Indeed, lying is more cognitive demanding than truth-telling, and this challenging cognitive process reflects itself in the human behavior, like reaction times (RTs) (43, 46) or mouse responses.

During this activity, participants are asked to answer truthfully or untruthfully to phrases shown on the monitor, using the mouse to click one between two alternative responses (“yes” or “no”) that appear on the screen. The analysis of the mouse trajectory highlights how false responses can be distinguished from the true ones. This statement is based on temporal and spatial dynamic parameters, such as the time to compute the response and the width of the mouse trajectory, as well as other kinematic parameters like speed and acceleration (47). Indeed, liars show wider and more erratic trajectories; they make more errors and take more time to compute their responses. On the other hand, truth-tellers are more rapid; they make fewer errors, and they are characterized by mouse trajectories straight to the responses.

The kinematic technique has been recently applied also to the detection of psychiatric disorders simulation. Monaro et al. (47) proposed to apply the kinematic analysis to detect the simulation of depression, catching mouse movements while the patient is engaged in responding to double-choice questions about depressive symptoms. The authors analyzed the difference in mouse trajectories between depressed patients and participants who were instructed to simulate a depressive disorder in order to gain a financial reward. Results demonstrate that this technique is able to detect feigned depression with an accuracy up to 96%.

Currently, there are no studies on crime-related amnesia in which kinematic technique was applied.

### SVT Procedures

One additional strategy to detect malingered amnesia in crimes consists of using forced-choice recognition memory tests, such as SVT (33, 48, 49). This is a well-known procedure used in civil courts to detect malingering, especially in mild traumatic brain injury. Its logic is as follows: if a patient is genuine, with an unfeigned impairment, he will not be able to choose the correct answer between the two stimuli; in this case, he should perform at chance level over many trials. On the contrary, malingerers usually select the wrong response deliberately and thus they perform significantly below chance. The most likely explanation for this performance is that the examinee knows the correct answer but decides not to choose it (49, 50).

The SVT procedure was also adapted to assess criminal defendants who claim to suffer from amnesia. The offender is asked to answer a series of questions based on facts or details linked to the crime deriving from police reports or third-party testimonies. Each question has at least two possible answers, one correct and the other incorrect but plausible. Generally, this information is presented orally or in written form on a computer screen, but alternatively, when visual material obtained during police inspections or images of the crime scene are available, it is possible to set up the test with such material [see, for example, Ref. (33)]. The visual presentation of images seems, in our opinion, preferable due to a reduced mnemonic load in terms of working memory.

Brandt et al. (48) proposed a first application of SVT in crime-related amnesia. They examined LG, a 64-year-old man charged with the murder of his wife claiming complete amnesia of the event. He was asked to freely recall a 20-item word list and then to attempt two-alternative forced-choice recognitions of each of the 20 words. LG freely recalled only four target words, and in a forced-choice recognition, he correctly selected only three of the target words. This was a performance worse than chance indicating that, at some level, he knew most of the 20-item words. It was suggested that he was feigning his anterograde memory deficit for violent crime.

Similarly, Denney (49) used SVT to evaluate crime-related amnesia in three cases of homicide. He collected a series of autobiographical information and data concerning the criminal events in order to create a questionnaire. Subjects were presented with written sentences where 50% of the cases were referred to real events occurring before the homicide. The remaining 50% of the phrases described a similar, but unreal, event. The task consisted of reading the sentences and saying whether they were true or false. All the subjects responded below the chance level, a result that indicates a voluntary strategy to avoid correct answers pretending memory loss of the criminal event. Recently, Jelicic (28) described the case of Randy, a 29-year-old man, accused of his girlfriend’s homicide, who claimed a complete amnesia of the murder. Reconstructing the crime scene, an SVT with 20 forced-choice questions with correct and incorrect but, plausible and similar, answers was created. Randy’s amnesia resulted in 14 incorrect answers out of the 20 items. According to binomial statistics, the probability that his response pattern was based on random guessing was <6%, indicating that there was a <6% chance that his amnesia was genuine. Those two elements led to converging evidence that Randy had feigned his amnesia for the stabbing. The court also found his amnesia claim not credible (28).

Again, in a multi-method approach study, Giger et al. (33) applied a forced-choice SVT in a mock homicide and found out only a low sensitivity of the procedure. The authors argued that the results are probably due to a few utilized numbers of items.

## Method

### Participants

Forty volunteers (20 female, 20 males; mean age = 24.5, *sd* = 8.27, range = 19–60 years) were recruited from the staff of the Bicocca University of Milan and the IRCCS Fondazione Caa Granda Ospedale Maggiore Policlinico of Milan. All participants had normal visual acuity and were screened for a history of psychiatric, neurological, or medical illnesses. The Ethics Committee of the Bicocca University of Milan and the Istituti di Ricovero e Cura a Carattere Scientifico (IRCCS) Fondazione Cá Granda Ospedale Maggiore Policlinico of Milan exempted us from initiating the practice of approval, considering the study as an observational type without drug use. Each subject participated in the study voluntarily, without remuneration. Before the experiment, all participants signed a disclaimer form in order to take part in the study in accordance with the guidelines of the University Committee. Two random groups, balanced for gender and age, were used.

### Procedure

The experiment took place in a single session and lasted between 30 and 45 min. It was designed in four steps: a) baseline thermography; b) mock crime; c) thermal imaging during kinematic test, and d) Symptom Validity Test procedure. At first, each subject entered a room and a baseline thermographic image was taken. The areas behind the eyes and to the sides of the nose corresponding approximately to the tear ducts were explored in detail. Lacrimal caruncle temperature (°C) was recorded by a certified technician (EH) using an infrared camera (NEC Avio TVS500; Nippon Avionics Co., Ltd, Tokyo, Japan). It was not possible to regulate room temperature and humidity, but they were relatively stable across all situations (minimum = 18°C, maximum = 22°C; mean = 20°C). Before every session, to define the radiance emission and to nullify the effect of surface reflections on tested participants, the same image of a Lambert surface was taken. Only images perfectly on focus were used. Grayess IRT Analyzer 6.0 software (51) was used to calculate the maximum temperature (°C) of a circular area traced around the caruncle area and of the body surface; this value was used for subsequent analysis.

**Figure 1:**
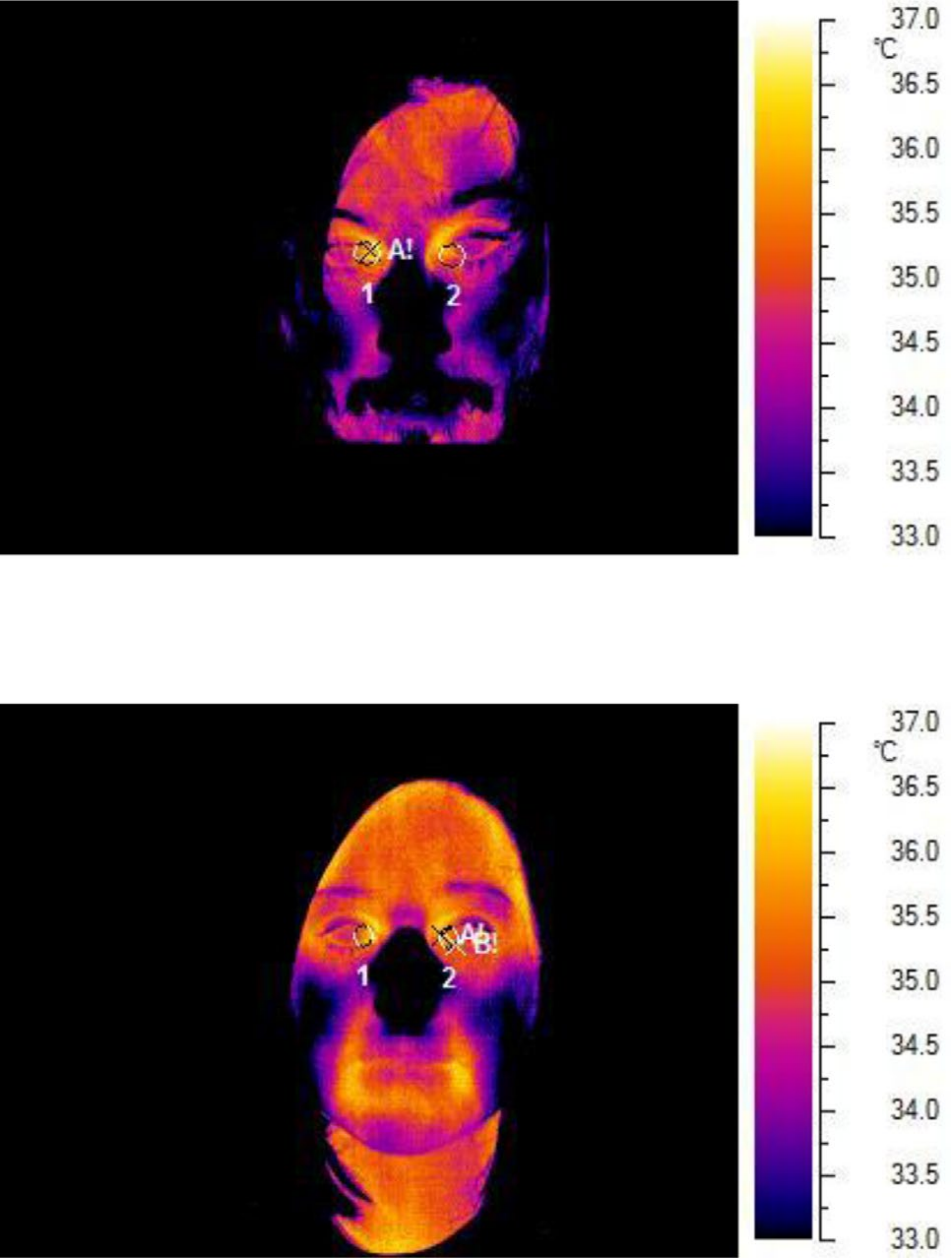
Thermal imaging.

**Figure 1** reports some examples of images obtained at baseline thermal imaging.

Afterwards, each subject was instructed with the following orders:

“You have to enter the room and pick up the knife on the table. Don’t worry, it is a fake knife which can harm no one! So, now go into the room. You will see a girl sitting at a desk with her back to you. This is a mannequin even if it seems real. There is a big box on the table, inside there may or may not be some money. Stab the girl violently in the back and check whether the box contained money or not. If it does, take it and run back to the room where you found the knife.”

**Figure 2:**
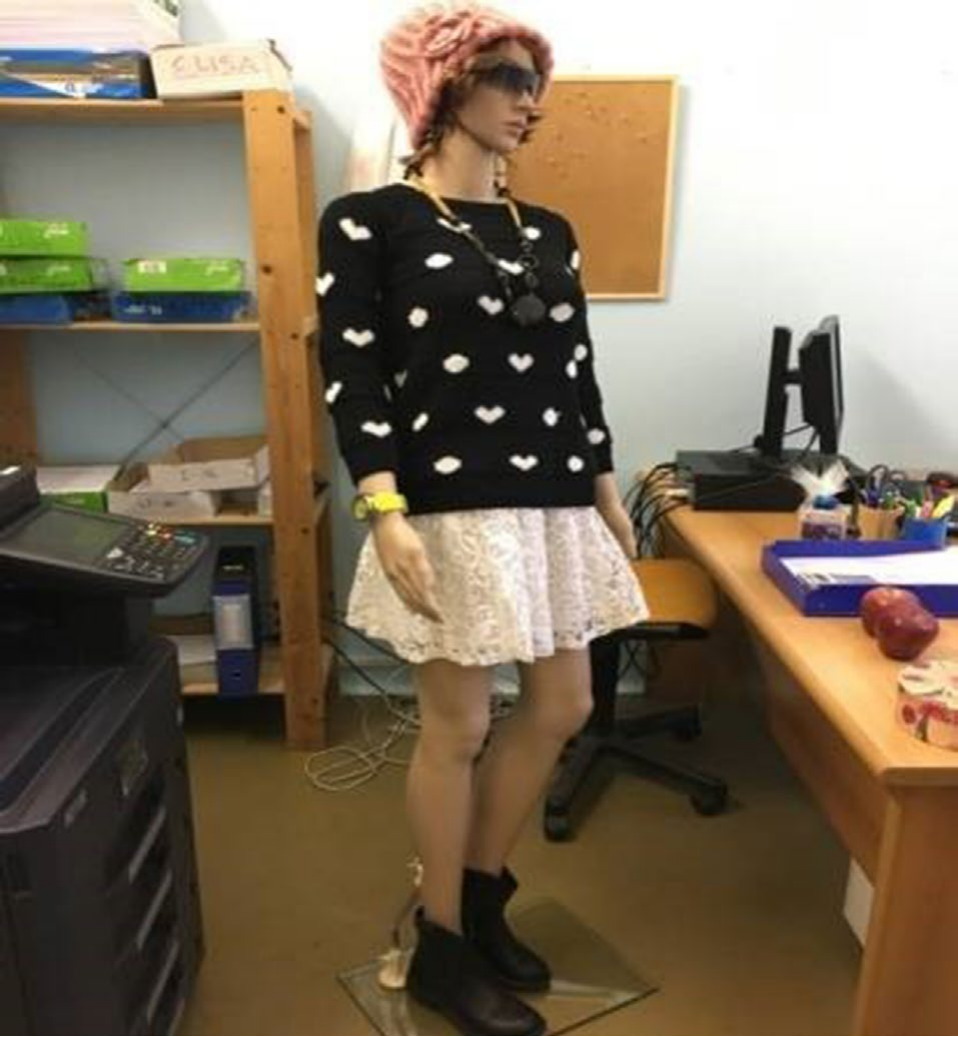
Mannequin used in the mock crime scene.

The mannequin was wearing a pink cap, black sweater with a white motif, a white lace skirt, and black boots. It also had sunglasses, earrings, a black necklace, and a yellow watch. Furthermore, the crime scene was composed of the following objects: two chairs, a desk, two red apples, a red rose, a fork, a black bag, a box containing jewels, and a computer. See **Figure 2** for a full representation of the crime scene.

Once subjects returned to the original room, they were assigned to two different experimental groups. The first group (*honest*; *n* = 20) was instructed to be honest and to perform accordingly in all the experimental phases. The second group (*naïve malingerers*; *n* = 20) was instructed to simulate a crime-related amnesia to avoid any criminal responsibility.

Immediately after, participants were asked to sit in front of a computer and to carry out a kinematic test, which analyzes mouse dynamics to detect deceptive responses. The details of the procedure have been described in-depth by Monaro and colleagues, in their paper regarding malingered depression (47). In this study, the mouse dynamics test was adapted to the analysis of crime-related amnesia. For example, test instructions for the honest group of our study were the following:

“The following questions concern the actual moment and the simulated homicide in question. Please answer all the questions honestly. If you are undecided about a question, mark the answer which you think is more correct. To answer, click ‘yes’ on top right or ‘no’ on top left of the screen. To see each question, click on ‘start’ at the center bottom of the screen. Some questions are composed of two phrases. To answer these questions, you should click ‘yes’ only if you agree with both phrases. To start the experiment press ‘shift’ on the keyboard.”

The task was programmed using *MouseTracker* software (52). Seventy-one sentences randomly appeared on the upper part of the computer screen and presented to the subjects. Participants were instructed to respond to each question by clicking on one of the two alternative responses (“*yes*” on the upper left or “*no*” on the upper right). The 71 stimuli included 16 types of sentences according to the complexity of the sentence (simple vs. complex sentences), to the required response (yes vs. no), and to the sentence topic (memory of mock crime vs. crime scene vs. test setting). Simple sentences (*n* = 15) contained only one piece of information related to the crime scene, the test setting or the amnesia symptoms (e.g., “*Do you remember the face of the mannequin?*”). Complex sentences (*n* = 56) were those containing two or more pieces of information—about the crime scene, the test setting, or amnesia symptoms—in the same phrase (e.g., “*Do you remember the face of the mannequin and are you wearing shoes right now?*”). Each piece of information in the phrase could be true or false, so a complex question required a “yes” response when both parts were true, whereas it requires a “no” response when at least one of the two was false (53). Simple and complex sentences regarded the memory of the mock crime (e.g., *“Do you remember what happened in the room?”*), the crime scene (e.g., “*Do you remember an apple and a bag in the room?*”), or the test setting (e.g., “*Are you wearing shoes right now?*”). In the **Online Supplementary Information**, the list of the sentences presented to the subjects is reported, including the information about the type of sentence and the expected response for each experimental condition.

Complex questions have been proved to be an accurate strategy to increase liars’ cognitive load and, as a consequence, to spot them. Indeed, responding to complex questions, the subject has to monitor the plausibility of more than one information and retain it in working memory to finally decide if the entire sentence is true or false. While truth-tellers can speedily carry out this sequence of mental operations, liars need more time to match the plausibility of each information with the lie they told (54). Responding to complex questions, liars have been demonstrated to have slower RTs and worst accuracy than truth-tellers (53).

The *MouseTracker* software recorded the spatial and temporal features of the mouse trajectory while the subject was responding (see **Table 1**). After computing the average value of all stimuli for each participant, the kinematic spatial and temporal features were used to compute statistical analysis. Finally, for each participant, we also calculated the average value of each feature for the 16 types of sentences. Then, these data were entered in machine learning (ML) models to predict whether a subject was honest or a naïve malingerer.

**Table 1:** Spatial and temporal features recorded by Mousetracker sotware.

	Feature	Description
**Temporal features**	Initiation time (IT)	The time between the appearance of the question and the beginning of the mouse movement.
	Reaction time (RT)	The time from the appearance of the question to the click on the response box.
	Maximum deviation time (MD-time)	The time to reach the point of maximum deviation.
	Velocity on *x*- and *y*-axis	The speed of movement of the mouse on *x*- and *y*-axis during the response.
	Acceleration on *x*- and *y*-axis	The movement acceleration of the mouse on *x*- and *y*-axis during the response.
**Spatial features**	Maximum deviation (MD)	The largest perpendicular distance between the actual trajectory and the ideal trajectory.
	Area under the curve (AUC)	The geometric area between the actual trajectory and the ideal trajectory.
	*x*-flip	The number changes in direction along the *x*-axis.
	*y*-flip	The number changes in of direction along the *y*-axis.

During the kinematic test, a second infrared thermographic image was taken for a comparison with the baseline image previously made. At the end of kinematic session, a self-filling computerized two forced-choice task (SVT) was administered to the subjects. It was composed of 25 questions concerning the mock crime scene. As reported in the introduction, SVT is one of the most extensively investigated measures for the detection of memory malingering and has been used in some studies to evaluate memory in a criminal forensic setting. A forced-choice SVT is based on the binomial theorem. It predicts whether, when an individual is asked questions with only two possible answers of equal probability, test results fall within a predictably random range and distribution. In particular, below-chance performance alone would be predicted by binomial values.

In our procedure, to make the SVT more sensitive, we modeled the task on the *Free and Cued Selective Reminding Test* (*FCRST*) (55). FCSRT is a measure of memory under conditions that control attention and cognitive processing. The aim is to obtain an assessment of memory unaffected by normal age-related changes in cognition. Differently from other memory tests, the FCSRT requires a study phase designed to control attention and cognitive processing in order to identify memory impairment, not secondary to other cognitive deficits. Subjects identify pictured items (e.g., grapes, vest) in response to category cues (fruit, clothing). In the test phase, subjects are asked to recall the items they learned (free recall). The category cues are used for a prompt recall of items not retrieved during the free recall to generate a score termed cued recall. The sum of free and cued recall is called total recall. Originally, this was composed of 12 figures of both living and non-living things. In our test, the 12 original images were replaced by six images of objects present in the crime scene and six distractors. All the images were subdivided into three cards with four items on each. The six objects in the crime scene were a fork, a pink rose, red apples, a necklace, a sweater, and a pink hat. The four images were placed in front of the participant who had to name all of them.

Participants were then asked to remember the 12 items. For the images they did not remember, a semantic cue was given (e.g., there was a flower). The procedure was carried out three times. Then, an interference task lasting for about 20 min was presented to the subject. As interference test, we used the “Deux Barrages Test” (56), which only implies attentional capabilities without overloading or stimulating memory recall. If naïve malingering subjects report a score below chance level, it is possible to state with good probability that they are malingerers.

## Results

All the participants followed the instructions and committed the mock crime. Data from the experiment were processed with IBM SPSS (version 24) and WEKA software (57).

### Thermal Imaging

We carried out two *t tests* on an independent sample to compare temperatures within the groups, one on the baseline condition and the other on the experimental condition. With regard to the baseline condition, the result demonstrates no difference between the two experimental groups (*t* = 1.675, *df* = 20.908, *p* = .109; see **Table 2**). Surprisingly, in the experimental condition, the lacrimal caruncle temperature decreases in the deceptive group compared to the honest one (*p* = .003). These data contrast with results obtained in previous studies [e.g., Refs. (38, 40)] where an increase in facial temperature was found in deceptive participants.

**Table 2:** Thermal imaging analysis with Levene's test.

		Baseline		Experimental group	
		Equal variances assumed	Equal variances not assumed	Equal variances assumed	Equal variances not assumed
Levene’s Test for Equality of Variances										
F		6.765		1.967	
Sig.		.014		.170	
T		1.741	1.675	3.159	3.210
df		32	20.908	32	31.326
*t* Test for Equality of Means										
Sig. (two-tailed)		.091	.109	.003	.003*
Mean difference		.27986	.27986	.37727	.37727
s.e. difference		.16078	.16706	.11943	.11753
95% confidence interval of the difference	Lower Upper	−.04764 .60736	−.06765 .62737	.13400 .62054	.13766 .61687

F, test statistic of Levene’s test; Sig., p value corresponding to Levene’s test; t, computed test statistic; df, degrees of freedom; Sig. (two-tailed), −value corresponding to the given test statistic and degrees of freedom; s.e., standard error.

t test was used to compare temperatures within the baseline condition and the experimental condition. In the experimental condition, a statistically significant difference between the two groups was demonstrated (*p < .001, p = .003).

### Kinematic Technique

**Figure 3:**
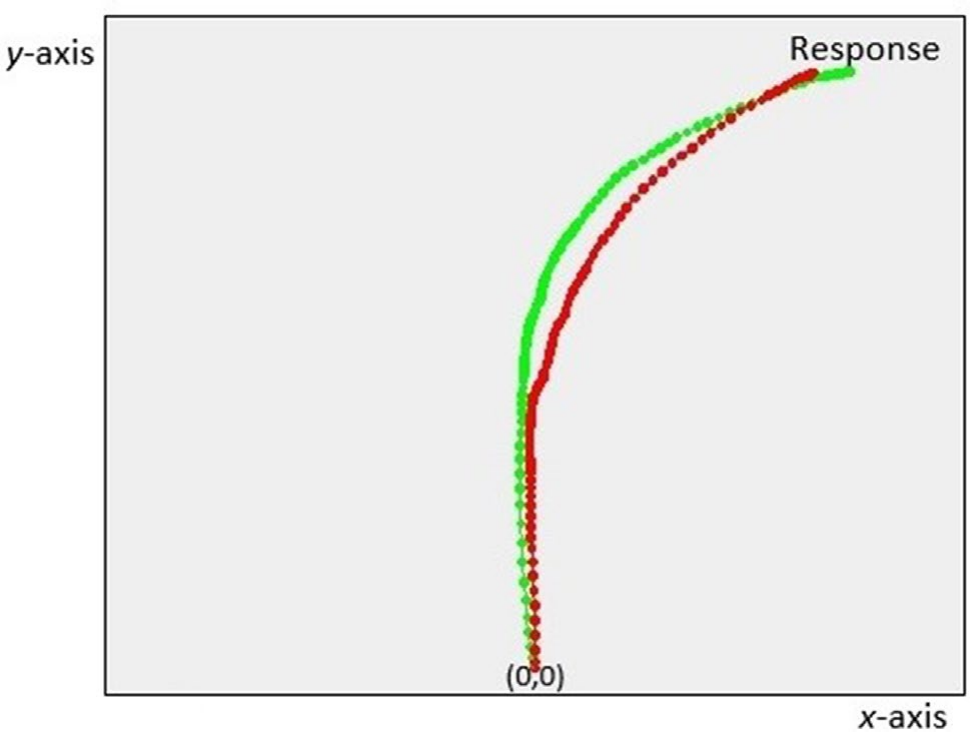
Average trajectories for liars and truth-tellers. The figure shows the average trajectories between the subjects to the expected YES and unexpected YES questions, respectively, for honest (in red) and for malingerers (in green). Expected and unexpected questions that require a YES response are those to which the malingerers lied. The values of the maximum deviation (MD), area under the curve (AUC), *x*-flip, and *y*-flip parameters for the two groups are reported. The gray area represents the difference in the AUC parameter between the malingerers and honest.

The kinematic results compared the responses of malingerers with honest subjects by averaging the responses to all stimuli across individuals. The analysis of kinematic spatial features, relative to the average of all stimuli to which subjects responded, shows that honest trace wider trajectory compared to malingerers [average honest maximum deviation (MD) = 0.69, *sd* = 0.21, area under the curve (AUC) = 1.46, *sd* = 0.72; average malingerers MD = 0.6, *sd* = 0.24, AUC = 1.35, *sd* = 1.09]. The average trajectories of both malingerers and honest are represented in **Figure 3**.

An independent sample *t* test was carried out on the 11 kinematic features [initiation time (IT), RT, MD, maximum deviation time (MD-time), AUC, *x*-flip, *y*-flip, velocity, and acceleration on *x*- and *y*-axis] obtained by averaging the 71 stimuli for each subject. To avoid the multiple testing problem, we applied a Bonferroni correction and the *p* value was set to .0045. Results showed a significant statistical difference between the two groups only for *MD-time* [*t*_(36)_ = −3.27, *p* < .0045, *sd* = −1.04].

Then, we analyzed the same 11 features by averaging the subjects’ responses to each of the 12 types of stimuli. Using a correlation-based feature selector (CFS), as implemented in WEKA software (57), we identified the features that are highly correlated with the dependent variable (honest vs. malingerers) while having low inter-correlation. Seven variables were selected and included as predictors within different ML models. The selected features are summarized in **Table 4**.

The trained ML algorithms were the following: Naïve Bayes, Random Forest, SVM, and K-nearest neighbours classifier (IBk). All the classifiers were trained using a 10-fold cross-validation procedure and reached an accuracy between 80% and 90% in distinguishing honest from malingerers. The accuracy for each classifier is reported in **Tables 3** and **4**.

**Table 3:** Description of the seven variables selected by the correlation-based feature selector (CFS) and entered in the machine learning (ML) models and their correlation with the dependent variable.

Feature	*r*_pb_
*x*-flip of simple sentences about the testing situation	0.44
MD-time of complex sentences about the memory of the mock crime and the testing situation requiring a no response	0.40
Velocity on *x*-axis of complex sentences about the memory of the mock crime requiring a yes response	0.44
Velocity on *x*-axis of complex sentences about the crime scene requiring a yes response	0.53
Acceleration on *y*-axis of complex sentences about the crime scene requiring a yes response	0.16
Velocity on *x*-axis of complex sentences about the crime scene and the testing situation requiring a yes response	0.63
MD-time of complex sentences about the crime scene and the testing situation requiring a no response	0.39

**Table 4:** Accuracy in distinguishing malingerers and honests obtained by four different ML classifiers using a 10-fold cross-validation procedure. Precision, recall, and *F* measure are also reported.

Classifier	Accuracy in 10-fold cross-validation	Precision	Recall	*F* measure
Naïve Bayes	89.7%	0.902	0.897	0.897
SVM	84.6%	0.862	0.846	0.845
Random Forest	89.7%	0.902	0.897	0.897
IBk	92.3%	0.924	0.923	0.923

IBk, K-nearest neighbours classifier; SVM, support-vector machine.

### Symptom Validity Test

Finally, in the *Symptom Validity Test*, a *t* test for independent samples showed a statistical difference between the two groups (*t* = 17.7; *df* = 31.22; *p* < .001). In addition, the results demonstrate that malingerers scored significantly below the chance level (*t* = −8. 159, *df* = 19, *p* < .001; *Z* = −1.84).

## Discussion and Conclusions

One of the main goals in crime-related amnesia is to find methods to detect malingering. Techniques of investigation are aimed to assist the court in evaluating the reliability of declarative proof that has been devised and perfected over a century. An increasing number of researches involving new lie detectors such as modern polygraphs, ERPs, thermal imaging, fMRI, kinematic analysis, facial analysis, or neuropsychological measures are applied today. Overall, studies have resulted in many promising findings. However, most of them highlighted the need of advances in the field with the consolidation of new methods driven by technical improvements.

The purpose of the present study was to investigate crime-related amnesia through the comparison of three new emerging methods (facial infrared thermography, kinematic analysis, and SVT) in a group of subjects invited to simulate, or not, an amnesia following a mock homicide. The results showed that kinematic analysis and SVT acquired significant accuracy in distinguishing honest from malingerers. However, thermal imaging results do not appear in line with those studies that reported more heat absorbed around the eyes when people lie.

With regard to SVT, the results of the present study clearly show better significance levels than those obtained by Giger et al. (33), who designed one of the first realistic mock crime experiments. Moreover, our data seem to be in line with earlier studies on real offenders (28, 49). It should be noted that in our SVT procedure, visual stimuli were used, along with controlling for the correct coding of the stimuli. In our opinion, this procedure and, above all, the implementation of visual material offer greater guarantee than the verbal version of the SVT in determining the veracity of crime-related amnesia.

To our knowledge, this is the first study to apply a kinematic analysis on an experiment involving crime-related amnesia. The results demonstrate the efficacy of this technique in detecting feigned amnesia, but they need to be further verified by additional studies.

Regarding infrared thermal imaging, we found that malingerers were slightly cooler than the honest subjects. A possible interpretation of this unexpected result is that such experimental conditions do not elicit a real emotional state. It should be noted that this is a measure of sympathetic nervous system and it differs from the other two techniques in which cognitive aspects, such as memory recall, are more prevalent. It has the advantage of being a contactless and non-invasive device able to record the spontaneous thermal irradiation of the face. We analyzed a specific region, the lacrimal caruncle, differently from most of the studies in the literature. Indeed, previous studies took into account the analysis of more distributed areas such as periorbital, supraorbital, and maxillary regions without focusing only on the lacrimal caruncle (36).

Animal studies suggest a relationship between the temperature of this area and the sympathetic nervous system (58). We examined this region to find a correspondence in humans. Our results show a little and non-significant decrease in the lacrimal caruncle temperature of the malingered group. Recently, Huggins and Rakobowchuk (59) applied a cold pressor test (CPT) and a muscle chemoreflex (MCR) to healthy subjects in order to activate the autonomic nervous system. No significant alteration in the temperature of the lacrimal caruncle was found. As the authors claimed, it is likely that changes in this region are more difficult to be detected using the infrared thermal imaging. Another possible explanation is that the human response is different compared to animals. The results of this study did not show an increase in the eye temperature between the baseline and the experimental condition. Since our aim was to find a very subtle variation when people lie, it is possible that the used infrared vision camera was not sensitive enough to detect such a change. A plausible interpretation of discordant results in literature is probably related to the complexity of the sympathetic nervous system in the lacrimal caruncle. It may be possible that, in this region, there is a different pattern of activation compared to the periorbital or supraorbital areas. Additional studies, with more refined thermal imaging approaches, are needed to clarify the activity of the autonomic nervous system through temperature changes in the human lacrimal caruncle. Moreover, the potential of this technique as a lie detector should be assessed more precisely.

In conclusion, the results of this preliminary study clearly highlighted the role of new lie detection methods in empirically supporting forensic professionals when discriminating between genuine and malingering crime-related amnesia. A multi-technique approach seems desirable and will be crucial in the translation of mock experimental to real single criminal case evaluation. In particular, future work, with defendant’s amnesia, will allow a more informed use of the three methods we have studied here.

## Ethics Statement

The Ethics Committee of the Bicocca University of Milan and the IRCCS Fondazione Ca Granda Ospedale Maggiore Policlinico of Milan exempted us from initiating the practice of approval, considering the study as an observational type without drug use. All subjects gave written informed consent in accordance with the Declaration of Helsinki.

## Author Contributions

Conceived the experiment: SZ and EP. Designed the experimental task: SZ, EP, MM, and AT. Data acquisition: EP, SZ and EH. Data analysis: SZ and MM. Data interpretation: SZ and MM. Drafting of the manuscript: SZ, MM, ES, TD and GO. All the authors revised the manuscript critically and gave the final approval of the version to be published.

## Conflict of Interest Statement

The authors declare that the research was conducted in the absence of any commercial or financial relationships that could be construed as a potential conflict of interest.

The handling editor declared a shared affiliation, though no other collaboration, with several authors MM, GO, AT at the time of review.

## Supplementary Material

The Supplementary Material for this article can be found online at: https://www.frontiersin.org/articles/10.3389/fpsyt.2019.00424/full#supplementary-material. The complete list of stimuli presented to participants during the mouse tracking task.
