# Supplementary material for: The Detection of Malingered Amnesia: An Approach Involving Multiple Strategies in a Mock Crime
Source: Front Psychiatry. 2019 Jun 17;10:424. doi: 10.3389/fpsyt.2019.00424 (PMC6589901; doi:10.3389/fpsyt.2019.00424)
Supplement: Supplementary file 1 [file DataSheet_1.pdf]

Supplementary Material

The detection of malingered amnesia: an approach involving multiple strategies in a mock crime

Stefano Zago*, Emanuela Piacquadio, Merylin Monaro, Graziella Orru, Erika Sampaolo, Teresa Difonzo, Andrea Toncini, Eugenio Heinzl

*** Correspondence:** Corresponding Author: stefano.zago@unimi.it

The complete list of stimuli presented to participants during the mouse tracking task.

The first column reports the complexity of each sentence (simple vs complex). The second column reports the content of the question (memory of mock crime vs crime scene vs test setting). Questions are reported in third column. Finally, the fourth column reports the expected response (yes vs no) separately for honest and naïve malingerers.

Complexity	Topic	Sentence	Expected response	
			Honest	Malingerers
Simple	Test setting	Are you sitting on a sofa?	No	No
		Are you in a room?	Yes	Yes
		Are you wearing shoes?	Yes	Yes
		Are you shirtless?	No	No
		Are you alone in the room?	No	No
	Memory of mock crime (amnesia symptoms)	Do you have vague memories of what happened?	No	Yes
		Do you have precise memories of what happened?	Yes	No
		Do you forget what you were doing in the room?	No	Yes
		Do you remember very little about the event?	No	Yes
		If you try, can you remember what happened?	Yes	No
		Do you remember why you entered that room?	Yes	No
		Are you ignoring what happened to the mannequin?	No	Yes
	Crime scene	Do you remember how the mannequin was dressed?	Yes	No
		Do you remember some details of the room?	Yes	No
		Is it difficult for you to remember the mannequin's face?	No	Yes
Complex	Crime scene + crime scene	Do you remember an apple and a bag in the room?	Yes	No
		Do you remember that the mannequin had a hat and it was serious?	Yes	No

		Do you know that you had to steal money but you found some jewels?	Yes	No
		Do you remember that you had to steal money and then to stab the mannequin?	Yes	No
		Do you remember whether in the room there was a closed window and a table?	Yes	No
		Do you remember that you found jewels and hit the mannequin?	Yes	No
		Do you know that the mannequin was on the right of the room and there was a window?	Yes	No
		As far as you remember, did you find any jewels and hit the mannequin?	Yes	No
		As far as you remember, were the jewels in a box, but you were looking for money?	Yes	No
		Do you remember that you saw the mannequin in the room and hit it violently?	Yes	No
		Do you know that the mannequin was sitting and wearing a hat?	No	No
		Do you remember that you wanted to steal money but also found some jewels?	No	No
		As far as you remember, there were also a bag and a dog in the room?	No	No
		As far as you remember, there were also a bicycle and a table in the room?	No	No
		Do you remember that you found some jewels that were behind the mannequin?	No	No
		Do you remember that you hit the mannequin and you took his hat?	No	No
		Do you know that the mannequin was sitting and you hit him with a knife?	No	No

		Do you remember that the mannequin was crying and had dark hair?	No	No
		As far as you remember, were apples on the table and did you steal one?	No	No
		Do you remember that there were chairs in the room and they were all red?	No	No
	Memory of mock crime (amnesia symptoms) + Memory of mock crime (amnesia symptoms)	Do you have vague memories of what happened and it’s hard for you to remember how the mannequin was dressed?	No	Yes
		Do you find difficult to remember the story and do you ignore what happened?	No	Yes
		Is the memory of the vent hazy and don’t you remember what there was in the room?	No	Yes
		Do you have flashes of what happened but you don’t know what you should do?	No	Yes
		Did you removed the incident and you can't remember it?	No	Yes
		Do you have memories about the event and do you remember how the mannequin was made?	No	No
		Don't you know what you did but you remember many details of the room?	No	No
		Do you know what you had to do in that room but can't remember what happened?	No	No
		Do you have any doubts about what happened and you perfectly remember what you found in the room?	No	No
		Did you removed what happened and you have precise memories of the room?	No	No
	Crime scene + test setting	As far as you remember, did the mannequin have a hat and are you sitting on a chair right now?	Yes	No

		Do you remember that there was a table in the room and are you in front of a computer right now?	Yes	No
		As far as you remember, were the jewels in a box and are you now in a room?	Yes	No
		Are you wearing shoes right now and do you remember that you stole money?	Yes	No
		Are you answering a questionnaire and did you find any jewels during the incident?	Yes	No
		Are you climbing a mountain right now and do you remember that there was a mannequin in that room?	No	No
		Do you remember that there was a dog in the room and are you in Italy in this moment?	No	No
		Do you remember that the mannequin was standing and are you shirtless right now?	No	No
		Is there someone with you in the room right now and was the mannequin sad?	No	No
		Are you now sitting on a couch and do you remember that you stole some money?	No	No
	Memory of mock crime (amnesia symptoms) + test setting	Are you in front of the computer right now and do you ignore what happened during the incident?	No	Yes
		Are you now in a room and do you have few memories of the story?	No	Yes
		Did you removed the incident from you memory and are you now responding with a mouse?	No	Yes
		Do you have only some flashes of what happened and are you reading questions right now?	No	Yes

		Are you wearing shoes in this moment and is it difficult for you to remember the story?	No	Yes
		Are you climbing a mountain and do you have vague memories of what happened?	No	No
		Did you removed the incident from your memory and are you in France right now?	No	No
		Is your mind clouded about the event and are you sitting on a sofa right now?	No	No
		Are you in the open country right now and doesn't you remember what you had to did in that room?	No	No
		Do you have only some flashes of what happened and are you now responding with a pencil?	No	No
	Test setting + test setting	Are you responding with the mouse and are you in a room right now?	Yes	Yes
		Are you in Italy and are you in front of the computer right now?	Yes	Yes
		Are you reading questions and is there someone else now in the room?	Yes	Yes
		Are questions written in red ink and you wearing shoes right now?	No	No
		Are you in the open country right now and is the door closed?	No	No
		Are you inside a building and are you now using a pencil?	No	No
